# Evaluation of Cracking Patterns in Cement Composites—From Basics to Advances: A Review

**DOI:** 10.3390/ma13112490

**Published:** 2020-05-29

**Authors:** Maciej Szeląg

**Affiliations:** Faculty of Civil Engineering and Architecture, Lublin University of Technology, 40 Nadbystrzycka Str., 20-618 Lublin, Poland; maciej.szelag@pollub.pl; Tel.: +48-81-538-4762

**Keywords:** cracking pattern, cracks, cement composites, concrete, image analysis, non-destructive testing

## Abstract

The structure and the development degree of a cracking pattern has a key impact on the durability of cement composites. This literature review focuses on the four most important aspects related to the evaluation of the surface cracking patterns, i.e., the process of formation, propagation and evolution of cracks into a branched system of cracks from the point of view of the fracture mechanics; the detection techniques of the cracking patterns on the surface of cement composites, where the tools of computer image analysis are the most used; parameters which can quantify the development degree and morphology of the cracks system; and also the influence of a cracking pattern on the functional features of cement composites. The studies described so far indicate the necessity of continuous development of this research area, because the knowledge of key relationships between the cracking patterns and functional properties of a cement composite is necessary to estimate the degree of material degradation. Researchers agree that the works carried out in the field of evaluation of the cracking patterns, to a large extent, contributes to the development of non-destructive testing methods in the field of cement composites technology.

## 1. Introduction

A cement composite is a conglomerate of aggregate grains combined into an artificial stone as a result of hardening of cement paste. This group consists of concretes—containing fine and coarse aggregate, mortars—containing fine aggregate and cement pastes, which in a sense can be considered as the micro-concretes. In this definition, the internal structure of a mature cement paste is composed of: relics of cement grains—as aggregate, gel-crystalline form of hydrated cement grains—as binder, free water remaining after the hydration reaction, as well as contractionary gel and capillary air pores of various moisture content [1]. The basic features of a fresh cement composite mix are its workability and potential strength. The latter feature manifests itself only after hardening but its value is pre-determined by the composition of the mixture and technology of its preparation. The mechanical strength first of all depends on the quality of the cement paste, i.e., the properties of the cement and the water/cement ratio (*w/c*). A fresh cement composite mix should contain enough cement and water to achieve the desired consistency on the one hand, and the hardened concrete on the other hand has the designed strength with filled aggregate cavities.

At the end of the 1920s, Brandtzaeg [2], who investigated longitudinal and transverse deformations of a concrete sample subjected to a uniaxial compression, found an increase in volume at close to destructive stress, which he attributed to an intensive increase in the number and size of the microcracks. Several years later the same phenomenon was observed by Yoshida [3]. For the next two decades, the phenomenon observed by the two aforementioned researchers was of little interest. It was only the growing problems related to safety and durability of reinforced concrete structures that drew the researchers’ attention again to the changes occurring in the structure of concrete under a short- and long-term load. The increasingly improved measuring equipment made it possible to develop new research methods, both direct and indirect, allowing to conclude on the destruction of structural bonds at various stages of loading. The first property that has been observed is the fact that in the case of strong aggregate grains, the cracks run through the contact surface of the cement paste-aggregate, the so-called Interfacial Transition Zone (ITZ). On the other hand, in the case of grains weaker than hardened cement matrix, these grains may be broken up. Today, this feature of the cement composite is considered crucial in terms of its durability and strength.

**Figure 1:**
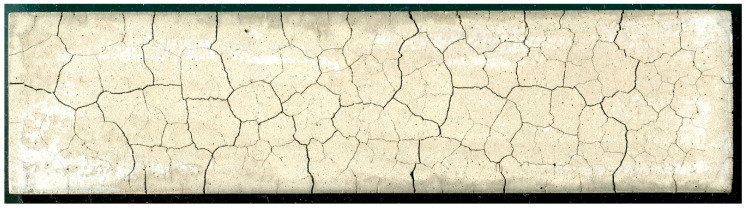
The cracking pattern on the surface of a cement matrix exposed to a temperature of 450 °C.

The disadvantage of cement composites is their ability to brittle cracking. Nevertheless, they are characterized by low plasticity, because the destruction under a static load occurs with moderately low total deformation. The brittle behavior limit is assumed to be a deformation of the order of 0.001 to 0.005 (relative value) at destruction stage, with high strength concrete being more brittle than normal strength concrete [1,4]. A sudden release of an elastic energy causes the local tensile strength of the cement matrix to be exceeded, resulting in a crack formation [5,6,7]. A characteristic feature of cement matrix and cement composites is the propagation and development of cracks under increasing load or duration of impact. The cracks, by joining and intersecting, form a network of cracks in the material, which is also referred to as the cracking pattern [8,9,10,11,12,13,14,15,16]. An example of the cracking pattern on the surface of a cement matrix is presented in Figure 1. In the cement composite technology, one of the key research areas is the analysis of the process of crack formation and development, because the formation of the cracking pattern is accompanied by a decrease in durability of the material. This is manifested by the deterioration of virtually all properties of cement composites, ranging from a decrease in mechanical strength, through reduced frost resistance, water resistance or resistance to aggressive chemicals. In the final stage of development of the cracking pattern there is a total loss of material cohesion, which results in its destruction.

The aim of the article is to provide a current state-of-the-art in the field of the analysis of the cracking patterns in cement composites. This research issue has a very high scientific potential and a great practical significance, but due to its multidisciplinary character it is sometimes difficult to implement. The analysis of the cracking patterns combines many fields of science, including the fracture mechanics, image analysis and technical aspects of the materials engineering—in this case, the cement composites technology. The scope of the work includes mainly consideration of the surface cracks. This literature review is divided into 6 main parts. The first section is an introduction to the topic of the cracking patterns in cement composites. The second section discusses the basics related to the process of formation and propagation of cracks in brittle cement materials. The process of evolution of single cracks into a complete, branched system of cracks present in the structure of the material is discussed. The analysis of the cracking patterns requires their extraction from the material structure. Since computer image analysis is definitely the dominant method of an automatic crack detection, Section 3 discusses the most important techniques used to extract cracks for further quantitative analyses. Section 4 discusses the most important and most frequently used quantitative parameters, which are used to describe the morphology and complexity of the cracking patterns. Then, in Section 5, the results of the most important studies on the influence of the structure of the cracking patterns on the functional properties of a wide range of cement composites are discussed in a synthetic way. The last section is a summary of the literature review, which indicates the most important problems and difficulties in quantitative analysis of the cracking patterns, and indicates possible further directions of research and development in this area.

What distinguishes this review from others of similar subject matter is the fact that the paper presents in a comprehensive way the problem of analysis of the cracking patterns in the structure of cement composites. There are reviews on image analysis itself, in which only methods of the digital cracks extraction are discussed. In such works there is a lack of information about the influence of the cracking patterns on the functional properties of cement composites. So far, no work has been recorded in which this subject has been discussed in a collective way, i.e., starting from the presentation of the mechanism that causes cracks and their transformation into a cracking pattern, through the techniques used for analysis, as well as quantitative parameters that can characterize the cracking patterns, to the most important thing from the point of view of the cement composites technology—i.e., how the cracking patterns affect the functional properties of this group of materials.

## 2. Causes of Cracks and Cracking Patterns in Cement Composites

Destruction of the cement composite is a consequence of its cracking, so it is important to consider this problem in detail. The actual strength of a cement composite or other brittle material is much lower than the theoretical strength, determined on the basis of molecular cohesion (strength of ionic or atomic bonds), and calculated on the basis of surface energy of a perfectly homogenous solid body (without internal damage). Theoretically, the strength should be approximately equal to tenth of the Young’s module, but practically it is much smaller. This state gave rise to the application of the Griffith’s theory [17] to describe the physical and mechanical properties of cement composites. Griffith was a pioneer of the fracture mechanics and he assumed that crystals always have defects that cause stress concentration in a small area, which is enough to locally exceed the theoretical fracture strength of the material. Starting from the energy balance of the process, Griffith determined the so-called crack propagation criterion. It gives the stress value which causes further cracking of the brittle material:

(1) $$\sigma_{k}=\sqrt{\frac{2EF}{\pi c}}$$

where:

E—the Young’s modulus of elasticity [N/m^2^],
F—the surface energy [N/m],
c—half the length of the crack [m].

Thus, for brittle materials that have a certain elasticity, the strength is dependent on the Young’s modulus, surface energy and a crack length. However, the energy required to produce a new surface due to cracking is actually higher than *σ_k_*. In order to take this into account, the Griffith formula has therefore introduced the fracture work *W* [J/m^2^] = [N/m], which, in addition to the surface energy, includes the plastic deformation of the material that accompanies the crack propagation:

(2) $$\sigma_{k}=\sqrt{\frac{2WE}{\pi c}}$$

The literature contains numerous studies on the applicability of Griffith’s theory to hardened cement matrix [18]. According to Mindess [19], the *W* for a cement matrix is in a fairly wide range of 7–14 J/m^2^. The determination of *W* itself is problematic, however, it is not necessary to determine *σ_k_*. For this purpose, it is sufficient to experimentally determine the critical stress intensity factor (*K_IC_*), which is the product of *W* and the Young’s modulus of the material. During the determination of *K_IC_*, the stress at which the material breaks is determined, which is equivalent to the moment when the crack begins to grow rapidly. The practical significance of the *K_IC_* lies in the fact that by knowing its value it is possible to determine the value of destructive stress depending on the shape and dimension of the crack and vice versa, i.e., by knowing the value of an operational stress in the element, it is possible to calculate the size of the critical crack at which it will break. In the light of the tests carried out so far, it appears that the *K_IC_* value for pastes made of Portland cement is in the range from 0.4–0.5 MN/m^3/2^ [20,21,22,23,24]. The current guidelines for the *K_IC_* testing of cement composites are contained in the RILEM Draft Recommendations [25].

**Figure 2:**
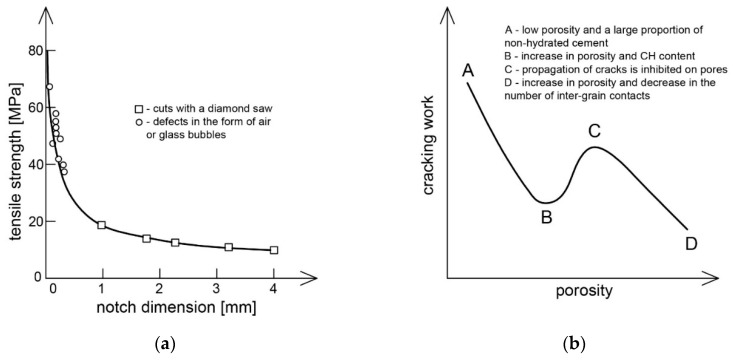
Dependency: (**a**) bending strength as a function of defect size (according to [34]); (**b**) cracking action as a function of porosity (according to [24]).

It is known that the physical and mechanical properties of cement composites are mainly determined by such factors as: total porosity, pore size distribution, the presence of material defects and the degree of structure variation. In the literature there are many studies [26,27,28,29] which indicate a strong correlation between the porosity of a cement composite and its strength. In this aspect, however, the pore structure is also of more importance, where it has been shown that the strength increases with decreasing pore dimensions [30,31]. Thus, the application of the classical fracture mechanics to the cement matrix means that the factor determining the strength will not be the total porosity, but the dimension of the largest gap—in this case the air pore [32,33]. An experiment conducted by Birchall et al. [34] proved that the bending strength of classical cement pastes lies on the curve determined from the Griffith’s equation (Figure 2a). The width of the gap (pore) was substituted for the dimension of the largest defect, naturally occurring in the material or artificially produced. On the other hand, the compliance of strength of cement paste with the overall porosity results from the fact that factors that reduce the overall porosity also reduce the dimension of the critical gap (pore) [35].

The dependence of *W* and *K_IC_* on the porosity of the cement matrix is complicated. Beaudoin [24] developed a qualitative course of this dependence, as shown in Figure 2b. Both of the above parameters depend to a large extent on the way the material is dried, i.e., ultimately on the humidity of the environment in which the cement matrix matures, as the effect of water on the strength of the cement paste is commonly known. The results of [20,24] show that a decrease in relative humidity of the environment from 100% to 0% results in an increase in *W* from 7 to 14 J/m^2^ and *K_IC_* from 0.20 to 0.46 MN/m^3/2^. Samples of cement paste soaked in water have a much lower tensile strength than dry ones. In the case of compressive strength, this feature increases slightly after drying the material [36,37]. Water, absorbing on the crack walls, shows the pushing pressure, which significantly facilitates the cracking process. On the other hand, the drying process removes the pushing water pressure on the crack planes near the crack limit. This was indirectly confirmed by an experiment conducted by Robertson and Mills [37]. They soaked the cement paste with organic solvents, which prevented significant reduction of tensile strength when moistened. Large molecules of organic solvents could not penetrate the microcracks, which prevented the pushing pressure.

The analysis of the effect of morphology of hydrated cement matrix on the cracking process was based on the hypothesis that portlandite crystals weaken its structure [38]. It was found that the cracks largely surround the areas where Ca(OH)_2_ crystals occur. However, in the light of other studies [39] it was found that the strength of portlandite is similar to that of the CSH phase. On the other hand, the reason that cracks form relatively easily and run along these crystals is the morphology of the CH crystals themselves in the transition zone and high porosity in ordinary concretes. Ultimately, this results in a reduction in concrete strength. A radical change in this situation occurs when the *w/c* ratio is reduced or microsilica is applied. In his considerations, Mindess [18] believes that empirical formulas combining porosity with strength can be used, but only for the classical cement matrix. In the case of significant modification of the structure resulting, e.g., from the presence of an additive or reactive admixture, or as a result of a hydrothermal treatment, the classical empirical formulas cannot be used.

The properties of concrete are mostly determined by the properties of the cement matrix. It is known that the hydrated cement matrix contains numerous discontinuities in the form of pores, microcracks and voids, which do not necessarily act as defects themselves. A defect can be, e.g., a crack in the grain that is associated with the presence of such a void, or it can be caused by shrinkage or insufficient adhesion. This is a natural situation in a cement composite, taking into account the heterogeneous structure of the material and the way the different phases combine. It has been confirmed [40] that pores in the cement paste are not the only possible critical defects of the structure. The application of the Griffith’s hypothesis assumes that first of all the voids in an unsegregated cement composite must be distributed at random, which is in line with the actual state of affairs [41]. Secondly, it is assumed that in the place where the defect exists there is a microscopic destruction, and the unit of material volume containing the weakest place determines the strength of the whole sample. Thus, each crack spreads over the entire section of the specimen subjected to a given stress or, in other words, a phenomenon occurring in an element is identified with the same phenomenon occurring in the entire volume. This occurs under the assumption of an even distribution of stresses and under the condition that the second consecutive weakest point in the material is not capable of carrying a stress *n/(n−1)* times greater than the stress at which the weakest point is destroyed, where n is the number of elements in the loaded section, each containing one defect. Thus, colloquially speaking, a cement composite is as strong as its weakest link.

Studies [42,43,44] indicate that in the ITZ and in the cement matrix itself there are very small cracks even before the load is applied to the cement composite. They are most probably the result of unavoidable differences in properties between the aggregate and hydrated cement matrix, combined with shrinkage and thermal stress. Microcracks are observed not only for cement composites of normal strength but also in unloaded concretes of *w/c* < 0.25, which mature in moist environment [45]. According to some studies [46], it is believed that the microcracks existing before loading are mainly responsible for low tensile strength of the cement composite. The microcracks are defined as cracks with a maximum width of up to 0.1 mm [1,46], which usually means the smallest dimension that can be followed by the naked eye.

The process of development of the microcracks under the compressive stress is already known. When a growing load is applied, the cracks remain stable to a load of about 30% of the limit load. Then the microcracks begin to propagate in terms of both length and width, and their number also increases. The stress at which the cracks develop depends on the *w/c* ratio of the cement matrix. This state is called as the stable crack propagation. As a result of further increase of the load up to a value between 70–90% of the maximum load, cracks are formed in the mortar. The cracks are then connected to the cracks caused by the breaking of adhesion and thus a certain system of continuous cracks is created, the so-called cracking pattern. This state is called as the state of rapid propagation of the cracks [42]. The stress level at the beginning of this state is higher in high strength concretes than in normal concretes. The increase in cumulative crack length is very large, while it is lower in high strength concretes [47]. The beginning of the state of rapid crack propagation corresponds to the point of discontinuity in volumetric deformations. If the load is fixed, the material can be destroyed as a function of time. However, as long as the cracks are stable, their presence is not harmful. This state is characteristic for the whole family of cement composites.

The structural heterogeneity of concrete is sometimes beneficial. For example, when the contact between coarse aggregate and cement paste is the site of local microcracks, it means that the presence of coarse aggregate grains prevents the opening of a single wide crack. These grains act as a blocking factor for the microcracks. The adhesion surfaces in the ITZ are formed at any angle to the direction of external force. As a result, local stresses differ significantly, upwards and downwards, from the nominal applied load. In the cement composite, cracks occur at each level of structural heterogeneity of the material [48,49,50,51]. Using an electron scanning microscope, the existence of a sub-microcracks was detected in the cement gel. However, there is no clear evidence that these sub-microcracks have a significant effect on concrete strength.

As shown above, the process of cracking and crack propagation is not a simple and unambiguous process. Very often, the formation of a crack may be initiated by one particular cause, and its development may be associated with a different cause [52]. Under certain physical conditions, the total width of cracks per unit length of the cement composite is usually constant. It is advantageous for the width of cracks to be as small as possible, which results in the fact that it is better if there are more cracks, but with a smaller widths than if there were fewer of them, but with a larger widths [12,13,15]. For example, the introduction of reinforcement controls shrinkage cracks by reducing the width of individual cracks, but does not change the total width of all cracks. The importance of the cracking process and the minimum width of the crack, which is considered significant, depend on the function of the structural element and on the exposure conditions of the cement composite. Table 1 summarizes the classification of cracks occurring in cement composites, together with the reasons for the formation and approximate time of occurrence of a given type of cracks.

## 3. Techniques for the Detection of Individual Cracks and the Whole Cracking Pattern

The first studies concerning the analysis of the morphology of cracks in cement composites were more qualitative than quantitative. Cracks were analyzed by means of a visual evaluation. This introduced great inaccuracies because the study was contaminated with human factor. Small portable measuring microscopes were started to be used for more accurate measurements, which were mainly used to determine the crack opening width in reinforced concrete structures [53]. It was only the development of digital technologies that initiated the development of research methods for the cracks analysis, as the computer image analysis was used for this purpose. The main problem of methods based on the digital image analysis is still the correct detection of cracks on the material surface. The reason for this is mainly a weak contrast of cracks to the remaining surface of the material. In the case of cement composites, cracks are visible as black or dark grey, and the natural shade of this group of materials is similar to grey. In recent years there has been a great development of digital testing methods for analyzing single cracks as well as the whole cracking patterns. This section discusses the most frequently used and at the same time most popular image analysis techniques, used in the analysis of cracks in cement composites, from the simplest in application to advanced segmentation algorithms.

### 3.1. Global Thresholding

The most basic and at the same time least complicated method of crack segmentation is the global thresholding [54,55,56]. It consists of determining for the whole analyzed image the global threshold value on the histogram, below which all pixels are interpreted as cracks and the rest are treated as background. This method is the least accurate of all methods of image analysis, because it can result in a very noisy image—cracks are very often integrated into the noise generated by the background. Depending on the complexity of the morphology of the analyzed surface, very often such an image is not suitable for analysis and drawing conclusions in terms of identification and evaluation of the cracking patterns. Currently, no analysis is performed using the global thresholding method, while the thresholding operation itself is popularly used at various stages of analysis, especially at an image pre-processing stage.

### 3.2. Locally Adaptive Thresholding

**Figure 3:**
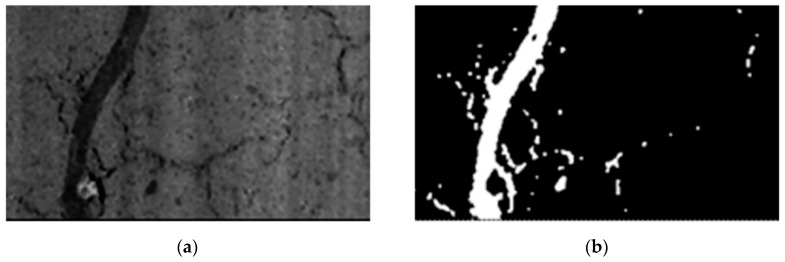
Example of the effect achieved by the locally adaptive thresholding: (**a**) original image; (**b**) after processing; based on [60].

Using this group of algorithms, the threshold is calculated individually for each pixel depending on the local statistics of neighboring pixels. Such statistics can be, e.g., variance, range or surface-fitting parameters. Finally, the threshold value is a function dependent on the position of the pixel, i.e., *T(i,j)*, where: *i, j*—coordinates of the pixel [56]. This method is much more useful when assessing whether or not there is a crack in the analyzed, local area. Tang and Gu [57] used the locally adaptive thresholding on the histogram, which was initially smoothed with the Gaussian filter. They obtained a good quality image showing the cracking pattern on the surface of the concrete road pavement, however, it was contaminated with small objects with non-linear characteristics. They used morphology operators such as the dilation operator and erosion operator to remove them. Tong et al. [58] developed software for cracks inspections for concrete bridges using adaptive thresholding elements in their algorithm. Liu et al. [59] developed a multistage method for identifying cracks in concrete assets, without any focus on specific application. In order to solve the problem of low contrast between the cracks and background, they proposed an image enhancement algorithm that uses the multi-scale guided filters with gradient information. The result was a well-contrasted image that uses the adaptive threshold segmentation to create a binary image representing the cracking pattern. Tests carried out on various images showing the real surfaces of cement composites showed the high validity and robustness of this approach. An example of using the method is shown in Figure 3.

### 3.3. Otsu Thresholding

**Figure 4:**
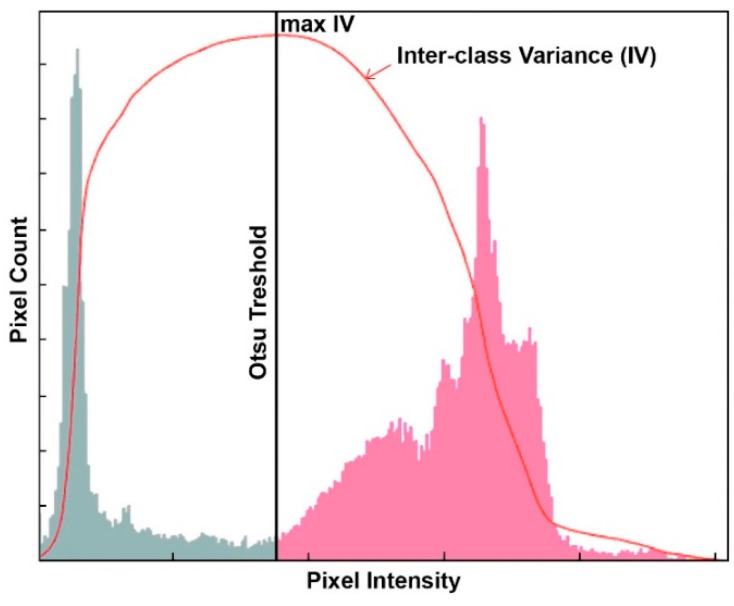
The Otsu method visualization.

The Otsu method is a very popular method, valued for its simplicity and efficiency. The algorithm was published in 1979 [61]. The aim of the algorithm is to binaryize the image, i.e., to convert the grayscale image to a binary image. The method is based on the histogram analysis and consists in minimizing the sum of the weighted variance of two classes (background and foreground objects), which is the same as maximizing the interclass variance. The method is particularly well suited for cases where the number of background and foreground pixels is similar. The idea of this method is shown in Figure 4.

**Figure 5:**
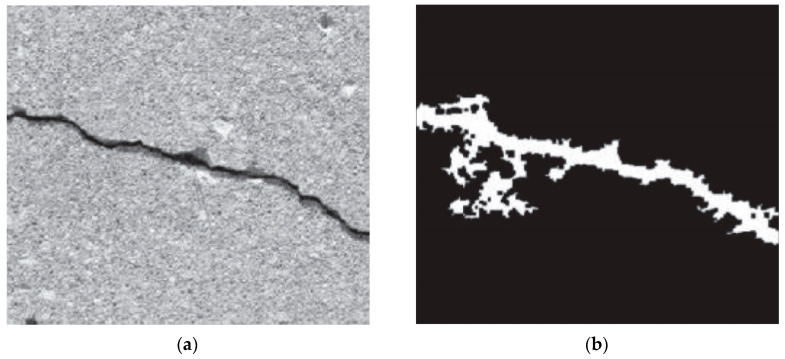
Extraction of cracks with Otsu thresholding: (**a**) original image; (**b**) after processing; based on [64].

Talab et al. [62] used the Otsu algorithm to detect major cracks on the surface of concrete structures. The original image was initially modified with a Sobel filter. The proposed method allowed for clear and accurate detection of cracks in images. Valenca et al. [63] developed an innovative method called the “MCRACK”, which aimed at automatic identification of the cracking patterns on the surface of cement composites, using digital image processing techniques. The whole procedure consisted of 6 stages. In one of the stages it was necessary to create a binary image which reinforced the discontinuities on the surface of the material. For this purpose, several algorithms of image binearization were compared, including the Canny edge detector, the Otsu method and the manual threshold. Binearization with the Otsu algorithm proved to be the most stable and gave the most accurate results, so it was decided to implement it in the final method. Hoang [64] proposed a way to improve the Otsu method (Figure 5) by earlier implementation of an image enhancement algorithm called the Min-Max Grey Level Discrimination. The whole procedure allowed for positive identification of cracks on the surface of cement composites, additionally analyzing the morphology of individual cracks, i.e., determining perimeter, area, width, length and orientation.

### 3.4. Genetic Algorithms

**Figure 6:**
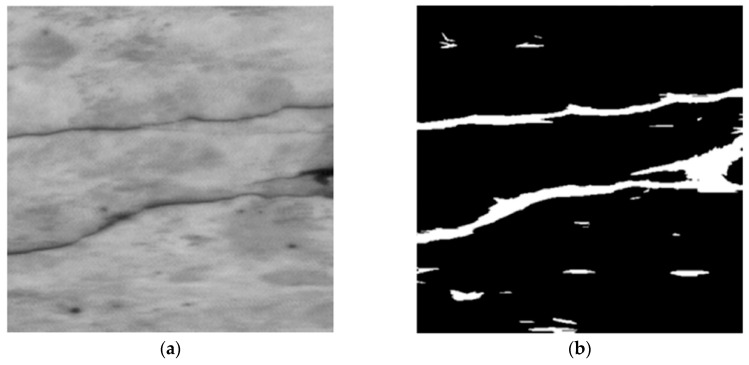
Extraction of cracks on a concrete surface using the genetic algorithm: (**a**) original image; (**b**) after processing; based on [69].

Genetic algorithms are applicable to many optimization problems. An algorithm is a kind of heuristics that searches the space for alternative problem solutions to find the best solutions. By definition, the pattern of genetic algorithms resembles the phenomenon of biological evolution. Nishikawa et al. [65] designed an algorithm based on genetic programming which created an image filter for cracks detection on the surface of cement composites. The developed algorithm was an improved version of the filtering technique proposed by Aoki and Nagao [66], who suggested that the technique developed by them could be used to solve many engineering problems. The algorithm proposed by Nishikawa et al. [65] presented robust performance for removing of noises from the image and for cracks detection. In his next work Nishikawa et al. [67] expanded the capabilities of the previously developed genetic algorithm to measure the width of crack opening and determine the spatial orientation of the cracks on the analyzed concrete surface. The crack opening width measured by means of the modified algorithm was in high agreement with the width measured manually, which confirmed the possibility of practical application of the developed method for health monitoring of concrete structures. Genetic algorithms were also used in the detection of cracks on road surfaces, where the input material was a monochromatic surface image [68]. An example of using the genetic algorithm is shown in Figure 6.

### 3.5. Fuzzy Logic Based Techniques

**Figure 7:**
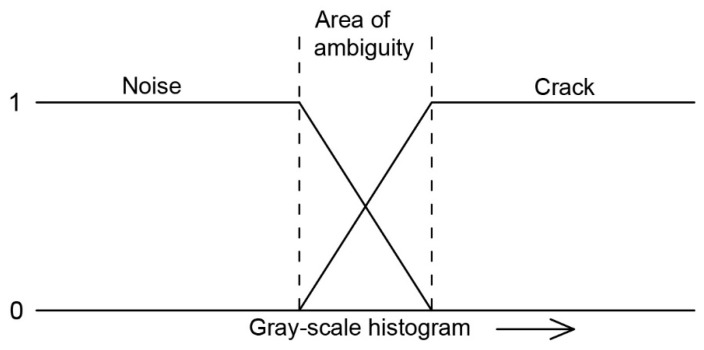
Model scheme of the fuzzy logic in an application to identify cracks on the surface of cement composites, where the input variable is the value on the gray-scale histogram.

Fuzzy logic is one of the multi-value logic, i.e., one in which more than two logical values are adopted. This term is closely related to the fuzzy set theory. In the fuzzy logic, a series of intermediate values extend between state 0 (e.g., false) and state 1 (e.g., true), which determine the degree of belonging of an element to a set. The fuzzy logic is very useful in engineering applications, because the classical truth/false logic cannot effectively deal with many ambiguous problems [70]. The problem of this type in the cement composites technology is precisely the digital identification of cracks using an image analysis. The problem of ambiguous identification of cracks on the surface of a cement composite using the fuzzy logic has been shown in Figure 7. The fuzzy logic techniques are often used in combination with genetic algorithms and artificial neural networks, which may result in the creation of an intelligent system with the ability to generalize knowledge in a given area.

Choudhary and Dey [71] used a fuzzy logic model in which they used “area” and “ratio” as input variables and the output variable was the “class” of the object. The class in this case was the pixel belonging to the crack or noise. Each variable consisted of two or more fuzzy subsets and corresponding trapezoidal membership functions. A total of 205 different images representing a cracked concrete surface were examined and it was found that the overall accuracy of the model was estimated at 90–94%. The fuzzy logic technique was also used to identify cracks on road pavements [72]. The whole developed method was based on the assumption that the crack is a continuous element and is darker on the histogram compared to its surroundings. The developed algorithm allowed positive identification of very thin cracks, even on very noisy pavement images. Yan et al. [73] developed an adaptive fuzzy image enhancement algorithm that effectively identified the cracking patterns on pavement surface.

### 3.6. Artificial Neural Networks

Neural networks are a very complex family of mathematical structures whose software or hardware models perform calculations or process signals through rows of processing elements called as the artificial neurons. The artificial neurons perform a basic operation on their input. There are different types of artificial neural networks, but their common feature is that their structure consists of neurons connected by synapses. Weights, or numerical values, are associated with synapses, whose interpretation depends on the model [74].

**Figure 8:**
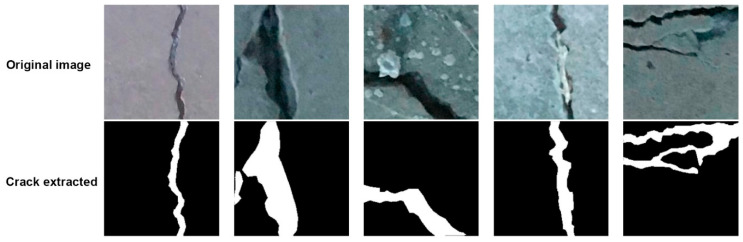
Results of crack detection by means of the artificial neural network, based on [79].

Lee and Lee [75] presented an integrated system consisting of three types of neural networks (image-based, histogram-based and proximity-based) to classify cracks on the concrete pavement surface. The system was validated on 124 actual pavement images. As a result of the analysis, it was concluded that the proximity-based neural network, despite the smallest computational requirements, is characterized by the highest accuracy in searching for the cracking patterns, which was estimated at about 95%. Bray et al. [76] developed a two-stage process of classifying cracks on the surface of concrete road surfaces using the artificial neural networks. In the first stage, based on the density and histogram, the artificial neural network divided into images with cracks and images without cracks. In the second stage, another neural network, after image segmentation, determined the type of cracks. The obtained results indicated 100% accuracy of the system in classifying images with cracks and 82% accuracy in classifying images without cracks. While the developed system gave good results in terms of cracks detection, it did not perform well in classifying the type of crack. Moon and Kim [77] developed an intelligent, automatic system for the cracks detection on concrete surfaces. The algorithm consisted of two main stages. In the first step, cracks were extracted from the digital image using filtering operations, the improved subtraction method and morphological operations. In the second stage, the cracks detection was performed by means of a backpropagation neural network. The whole algorithm was trained on 105 images of cracked concrete surfaces and then validated on another 120 images. The developed algorithm was characterized by a very high recognition rate, amounting to 90% for crack images and 92% for non-crack images, respectively. The authors emphasize the simplicity of the application of the algorithm, which makes it possible to assess the occurrence of cracks by non-expert inspectors. The versatility of programming artificial neural networks made it a very popular technique for detection of the cracking patterns, where further examples of application can be found in [78,79,80,81]. An example of the effect of identifying cracks on concrete surfaces with the implementation of artificial neural networks is shown in Figure 8.

### 3.7. Dijkstra Algorithm

In theory, the algorithm is used to solve the problem of the shortest path, i.e., the problem of finding the shortest connection between given vertices in a weighted graph. The operation of the algorithm is based on the fact that when analyzing a given graph with a distinguished vertex there are distances from the source to all other vertices. In a very simple way, the algorithm can be modified to look only for the shortest path to one fixed vertex, interrupting the action when reaching the target vertex or transposing the graph’s incident table. The feature of the Dijkstra algorithm is that it finds all the shortest paths between the selected vertex and all the others, while calculating the cost of passing each of these paths [82]. Using the Dijkstra algorithm is a very effective method to predict the occurrence of cracks in a given area, when there are cracks discontinuities due to a contamination of the digital image or local crack bridging is present.

Amhaz et al. [83] proposed an original method of automatic crack detection on digital pavement images, which main assumption is the location of minimal paths within each image. The developed minimal path selection algorithm consists of three main parts, i.e., endpoint selection, minimal path estimation using the Dijkstra algorithm and minimal path selection. The algorithm is supplemented by two post-processing steps, i.e., elimination of artifacts, and crack width detection. The developed algorithm was validated on real cracked pavement images and compared with five other crack extraction methods. The proposed algorithm automatically provided very robust and accurate results in a wide spectrum of situations, which is undoubtedly its advantage. Gunkel et al. [84] developed a procedure for automatic micro-crack detection that uses a shortest path algorithm. The method is very effective for the identification of microcracks that occur in a plastic deformation environment, where separation between cracks and plastic deformations is difficult. In the first stage of the method, cracks are detected as combined cluster systems, consisting of pixels with a gradient value below a certain threshold value. Then, the crack paths are identified with the Dijkstra algorithm as the longest shortest paths through the darkest areas of the cluster system. The developed method was applied to more than 2000 digital images of cracked surfaces. However, the procedure has some limitations because it assumes that the crack is only one path. The method is not suitable for identifying tree-like cracks.

Many researchers note [83,84,85,86] that using the Dijkstra algorithm to solve the problem of cracks detection on the surface of cement composites requires a very large amount of computational effort, which only highly specialized machines can cope with. It is necessary to further search and modify the algorithms that deal with the issue of the minimal path selection.

### 3.8. The Bayesian Classifier

One of the most effective methods of extracting the cracking patterns from a digital image of the cracked surface of a cement composite is the use of machine learning algorithms. One of the most popular algorithms used for this purpose is the Bayesian classifier, which is a simple probabilistic classifier. The classifier is based on the assumption of mutual independence of predictors (independent variables). They often have no relation to reality and therefore the classifier is often referred to as the Naive Bayesian classifier. In this case, the probability model can be derived using the Bayesian theorem, which binds the conditional probabilities of two mutually dependent events. Depending on the type of accuracy of the probability model, the Bayesian classifier can be effectively taught in a supervised learning mode. In many practical applications, the estimation of the parameter for the Bayesian classifier occurs using the maximum probability method “a posteriori”, which is a kind of paradox because the classifier can be used without believing in the Bayesian theorem. A characteristic feature of the classifier is that the classification is valid as long as the correct class is more likely than others. Despite its naive design and many simplified assumptions, the Bayesian classifier is very effective in real situations [87].

Schmugge et al. [88] developed an automatic crack identification method for use in concrete and steel construction elements of nuclear power plants. In this case the Bayesian classifier was used to classify whether a pair of line segments belong to the same segment or not. These linear segments were a segment of the cracks. Then, certain geometrical features of the line were determined for each segment, e.g., distance, orientation and similarity. The tests carried out on real images using the developed method showed an improvement in the ability to identify cracks by 38% compared to the reference methods. Hutchinson and Chen [89] proposed an automatic system for the detection of various types of defects in concrete, not only cracks but also cavities. Their procedure is based on the Bayesian decision theory. The detection of a probable crack is based on the implementation of two algorithms, i.e., the Canny and the fast Haar transform. The Bayesian classifier, on the other hand, decided whether the identified area in the image, which may be a crack, is really a crack. The advantage of the developed method is a quite accurate localization of concrete surface damage, with a small calculation effort. Valenca et al. [90] went one step further and created a powerful tool called “SurfCrete” for analyzing concrete surfaces. With this method, it is possible to classify an area into different classes, e.g., crack, biological corrosion, exposed aggregate grain or repair mortar. In their work they used two types of classifiers, i.e., the Bayesian classifier and the Multi-Layer Perceptron. Each of the classifiers was taught on a fragment of the surface showing a given morphological change of the concrete surface. The effect of the method is the possibility to create a map of concrete surface damage. After validation of the method, the accuracy of approx. 94% was obtained on the real concrete surface images. The Bayesian classifier is also popular in cracks detection systems in road surfaces [91,92].

### 3.9. The AdaBoost Classifier

The AdaBoost classifier is another tool used by machine learning algorithms. It is one of the basic algorithms for boosting, which is a method by which a large number of weak classifiers can be used to get one better. In this case, a weak classifier is one that is relatively simple and can classify test data with an efficiency of more than 50%. The classifier works in such a way that in subsequent iterations it trains and then measures the error of all available weak classifiers. In each subsequent iteration the importance of badly classified observations is increased, so that the classifiers pay more attention to them. The algorithm can significantly improve the quality of classification, but this improvement is only observed when weak classifiers are used as components. When more complex classifiers are used, the use of the AdaBoost algorithm does not lead to a significant increase in effectiveness [93].

Cord and Chambon [94] used the AdaBoost classifier to classify defects on road surfaces. The whole method is based on an appropriate selection of linear and non-linear filters depending on the texture of the analyzed image. In this case, the task of the AdaBoost classifier is to choose the most optimal set of filters to extract cracks in a particular case. A number of textural descriptors are used to learn the classifier. The obtained results were compared with the results of the methods already described in the literature and high efficiency of the developed method was noticed. Prasanna et al. [95] developed an automatic method for the cracks detection on bridge deck surfaces, which they called as the STRUM. The use of machine learning algorithms allowed to eliminate the need for manual definition and tuning of threshold parameters. The developed method uses the robust curve fitting to locate potential cracked areas. Three types of classifiers were used within the algorithm, i.e., the AdaBoost, support vector machines and random forest. The procedure allows to develop the crack density maps in the analyzed area. The accuracy of the algorithm was estimated at about 90%.

### 3.10. Summary of Techniques for Identifying Cracks and Their Accuracy in the Literature Review Works

The authors of the works, which have been included in the literature review, stress the universality of individual methods, as they can be applied to virtually any engineering problem aimed at identifying surface linear structures. The presented crack identification techniques have been developed mainly to apply to the surface of cement composites, practically in every application, i.e., bridge structures, reinforced concrete tanks, floor and wall structures, structures in nuclear power plants, dams, etc. The second group of application that stands out is road surfaces, both concrete and asphalt.

Table 2 synthetically lists the types of techniques used to detect surface cracks in cement composites embedded in various types of structures, with references to literature items covering the scope of this literature review. In most cases the developed cracks detection algorithms are often a combination of many techniques, so the list also includes those methods which are not discussed in detail. In addition, the levels of accuracy of cracks identification obtained by individual researchers are presented, based on the literature survey.

Apart from methods closely related to the computer image analysis, there are also other methods of cracks detection, e.g., infrared thermography, x-ray tomography or acoustic emission.

## 4. Parameters to Describe the Morphology of the Cracking Patterns

The cracking pattern on the surface of a cement composite is a kind of surface structure. The morphology of this structure changes depending on many technological factors, such as the composition of the composite, the factor causing the cracking process, the duration of load, etc. Scientists have always wondered what reliable parameters from already existing ones can be used to quantify the cracking pattern. An alternative was to look for and define new quantitative parameters that would be used for this particular purpose. This section describes the most important and most frequently used quantitative parameters used to describe the morphology of the cracking patterns.

### 4.1. Opening Width of the Cracks

The crack opening width is one of the simplest parameters to measure, as measurements can be taken locally using portable optical microscopes. However, such a measurement is burdened by the error of the human eye. Greater measurement accuracy is achieved by using a digital measurement, on a previously prepared image of the tested surface. This parameter is also of great practical value, since practically every aspect of durability of cement composites depends on the opening width of cracks. Due to the simplicity of the measurement, it is very often used to measure the width of cracks opening as a supplementary test to the main research thread concerning the cracking patterns. It is also valuable to give the maximum crack width, because in the case of a cracked cement composite, its mechanical strength is largely dependent on this parameter.

Wagner et al. [96,97] proposed a classification of the measured crack width distributions as the crack width polygons. Cracks of particular widths are accumulated in percentage. The graph of the crack width polygons allows very quickly to determine the percentage of cracks with a width, which is below a certain limit. This parameter allows to characterize the whole of the cracking pattern, because it is physically very similar to the construction of the particle size distribution curves—in case of analyzing aggregates for cement composites.

### 4.2. Length and Orientation of the Cracks

The length of the cracks or the entire cracks system, as well as the orientation of the cracks, are one of the basic parameters for determining the properties of a cracks. It is relatively easy to measure when implementing computer image analysis into a research program. The combination of these two properties, which characterize the cracking pattern, can provide a lot of information, e.g., regarding the direction of shrinkage or swelling of the cement composite, the direction of the aggressive agent action, etc. The orientation of cracks on a degraded composite is very important for the prediction of its mechanical properties, as well as for predicting the direction of further development of the cracks network.

In his research, Szeląg [12,13,14,15,16,98] introduced a modification of the crack length measurement, by measuring the length of the perimeter line of the area which is limited by the cracks. This approach applies to the analysis of cracks that form closed areas called as the clusters, on the surface of the material. The clusters are formed only when there is a strong volumetric cracks propagation, e.g., in case of a thermal load.

### 4.3. The Crack Density

In the early 1990s, Mobasher et al. [99] defined a new parameter to describe the morphology of the cracking patterns, which is commonly used today, namely the crack density. This parameter was defined as the ratio of the average length of the crack to the area of the surface under analysis, according to the equation:

(3) $$L_{A}=\frac{2\bar{L}}{a^{2}}$$

where:

$L_{A}$—the crack density [mm^−1^],
$\bar{L}$—the average crack length [mm],
$a^{2}$—the area of the test section [mm^2^].

A slightly modified version of the crack density, i.e., the parameter expressed as number of cracks per meter [13,96,100], is also encountered in the research. In this version, the crack density is more convenient to use, because in order to get the result it is necessary to count the number of cracks crossing the test line, and relate the result to the situation when the test line would be 1 m long. In this case, with the crack density it can be very easily calculated the crack spacing, which is a size that symbolizes the average distances between the cracks.

### 4.4. The Crack Area

The crack area is a surface parameter and determines which area is occupied by cracks. This parameter is used in two versions, i.e., as an absolute value expressed, e.g., in mm^2^, and as a relative value in relation to the total area of the area under analysis, then often the crack area is given as a percentage value. Compared to the previously mentioned parameters, the crack area is more difficult to determine because it requires more complex digital image processing techniques.

Similarly as in the case of measuring the crack length, also in the case of the measurement of the crack area, Szeląg [12,13,14,15,16,98,101,102] applied a modification consisting in the measurement of the area which is limited by the cracks.

### 4.5. The Fractal Dimension

Very often such local parameters as its length or width of the opening are insufficient to describe the complexity of the cracking pattern. Thus, in recent years there has been a growing interest in using the concept of fractal dimension for this purpose. The use of fractal geometry to characterize the cracking pattern has many advantages compared to the use of Euclidean geometry. In order to use this dimension, it is necessary to assume that the structure created by the cracking pattern is in fact a fractal, i.e., it has certain characteristics such as [103,104]:

- formlessness—an unambiguous shape is impossible to determine,
- description by a recursive relationship, not a mathematical formula,
- self-similarity—an isolated fragment resembles its larger whole.

Looking at these assumptions, it is not difficult to resist the impression that the cracking pattern visible on the surface of cement composites fully meets these assumptions. In such a case, in the fractal geometry, it is the fractal dimension that determines how densely a fractal fills the metric space it is in. In the studies on the cracking patterns described so far, the fractal dimension is used as a stand-alone parameter, but often also serves as a component of an extensive index for evaluating the degree of material degradation [105].

### 4.6. A Summary of the Use of Individual Parameters to Describe the Morphology of the Cracking Patterns

Table 3 summarizes the parameters used to describe the morphology of the cracking patterns. The list includes works that have been included in this literature review.

## 5. Influence of Morphology of the Cracking Patterns on the Properties of Cement Composites

It is well known that cracks in cement composites are a defect in the material. Practically all functional properties of a cracked material deteriorate. The most important results of the research published so far concerning the influence of the structure of the cracking patterns on particular properties of cement composites are presented below in a synthetic way. The main focus is on performance at elevated temperatures, shrinkage and drying, water permeability, mechanical strength and chemical corrosion.

### 5.1. Performance at Elevated Temperatures

Mobasher et al. [99] examined the cracking process in cement composites, with different polypropylene fibers content, using an acoustic emission technique and quantitative image analysis. The morphology of the crack system was investigated for samples loaded to specific strain magnitudes. For this purpose, the crack density, length and spacing were determined. An average crack density of 1.27 mm^−1^ was obtained. It was observed that as the strain level increases, the formation of new cracks is accompanied by decrease in the crack spacing. Thus, the presence of polypropylene fibers in the cement composite resulted, first of all, in the necessity to supply more energy to the system so that the existing cracks could further propagate; secondly, an increase in the degree of cracks and microcracks dispersion in the material, which undoubtedly improved the mechanical properties of the composite.

Xu et al. [9] analyzed the morphology of the cracking patterns of concretes subjected to a high temperature. The subject of the study were concretes differing in the *w/b* ratio (water/binder) and fly ash content. A visual imaging technique was used to study, among other things, the crack density. It was noted that the crack density increased almost linearly up to 650 °C. There was a rapid increase in this magnitude between 650–800 °C. The degree of cracks dispersion increased as the fly ash content increased. Smaller *w/b* values were accompanied by a smaller number of cracks, however, the resulting cracks were characterized by a larger opening width and length, compared to concretes with a larger *w/b* ratio. It was found that a decrease in tensile strength with an increase in thermal load is identical with an increase in the crack density.

The studies carried out by Kim et al. [120] concerned the influence of thermal load (in the range 25–1000 °C) on the properties of cement paste. Among others, the 3D X-ray Computer Tomography was used to determine the change of pore structure and to evaluate the process of initiation and spatial configuration of thermal cracks, as a function of the temperature acting. It was observed that at temperatures higher than 900 °C a massive cracking network is formed in the edge zones of the material. This effect was considered to be the main reason for the occurrence of explosive spalling, which is one of the most dangerous phenomena accompanying cement composites exposed to the fire.

Magalhaes et al. [100] examined the mechanical response of a cement composite with PVA fibers. The samples were preheated at 90–250 °C. Next, uniaxial tensile, bending and compressive strength tests were carried out, during which the crack width and the crack density were measured. The studies showed that the crack density takes less and less values at the same level of deformation as the preheating temperature increases. In the case of the crack width, the inverse relationship was observed. The maximum crack widths were in the range 100–220 μm. The conducted research confirmed that in terms of mechanical performance, the more favorable situation is when there are more cracks in the analyzed area, but they are of smaller widths.

Szeląg evaluated the influence of microsilica [14,98,102], metacaolinite [16,101], polypropylene fibres [12] and multi-wall carbon nanotubes [13] on the process of formation of the cracking patterns in cement pastes, under thermal load, in the range 200–250 °C. The influence of the shape and size of the sample on the geometric characteristics of the cracking patterns was also investigated [15]. To describe the morphology of the cracking patterns, the following parameters were used: the area and perimeter of the cluster, the width of the crack opening and the crack density. It was found that modification of the cement matrix with the addition of microsilica causes densification of the crack network. There are more cracks, but they have a smaller opening widths, which has a positive effect on mechanical properties. In case of modification with metacaolinite, a decrease in the number of cracks formed with comparable widths of their opening in comparison to unmodified cement matrix was observed. The use of polypropylene fibers resulted in the reduction of area and perimeter of the clusters, with a negligible effect on the opening widths of the cracks. The polypropylene fibers melted due to thermal load and the cracks forming the cracking pattern were largely contained in cavities left by the fibers. In case of modification of the cement matrix with carbon nanotubes a drastic reduction of cracks system density was observed, with a simultaneous high increase in crack widths. It had a negative effect on mechanical performance. However, the effect observed was more related to the phenomenon of cement matrix foaming caused by the way carbon nanotubes were introduced than to their presence itself.

### 5.2. Shrinkage and Drying

One of the first preliminary observations on the influence of the drying process on the characteristics of the cracking pattern were made by Bazant et al. [110,111]. Concrete and cement pastes were the subject of research. They noticed that as a result of the drying process, a network of parallel cracks is formed, whose spacing and width of opening is proportional to the depth of penetration of drying. With typical test samples, most cracks are too thin to be observed with the naked eye. It was also found that the characteristics of the cracking pattern predominate in the direction of the development of discontinuous microcracking rather than in the direction of continuous macrocracks.

Wang et al. [106] studied plastic shrinkage behavior in cement composites with the addition of fly ashes and various types of dispersed fibers. The structure of air pores using a mercury-intrusion porosimetry and the cracking pattern using the image analysis were studied. The maximum crack opening width was measured as well as the crack area. It was observed that the addition of dispersed fibers generally increases the number of large pores in cement paste, which affects both bleeding behavior and crack distribution in the material. It was found that, depending on the type of fibers and their properties, the crack area can be reduced by 30–40%, with 0.1% (by volume) of fiber addition. It has a very big influence especially on the mechanical properties of the cement composite in the early stages of maturation.

Plastic shrinkage behavior of cement pastes with the addition of alkali-resistant glass fibers was studied by Bakhshi and Mobasher [107]. The addition of glass fibers in the amount of 0.06%, 0.11%, 0.17% and 0.23% by volume was studied. The tests were carried out in low-pressure conditions, and additionally the sample size, *w/c* ratio and conditions of initial care were also a variable factor. The morphology of the cracking pattern was analyzed using the image analysis. It was found that the drying process can be divided into two main stages, i.e., stage I—constant drying rate period, and stage II —falling drying rate period. The research confirmed that by using glass fibers it is possible to control the process of formation and propagation of cracks during the maturation process of the cement matrix. While it was not observed that the presence of fibers had an impact on the cracking pattern in the stage I, in stage II the presence of fibers reduced the diffusivity values. Scientists concluded that the reason for this was the bridging effect of the glass fibers. The results showed that in the case of 0.11% and 0.23% of fibers addition, the areal fraction of cracks was reduced by 22% and 61%, respectively. The maximum crack opening width was also reduced by 47% and 71%, respectively, compared to classical cement paste.

Bisschop and Wittel [8] conducted a study to evaluate the morphology of the cracking patterns in classical cement paste with the *w/c* = 0.5. The study determined the effect of sample thickness and methods of drying on the crack density, crack length and the crack penetration depth. The latter parameter was quantified using a fluorescent resin impregnation technique. It was found that for samples < 14 mm thick, the single-sided drying resulted in lower values of the crack penetration depth (15–20% of the sample thickness), compared to double-sided drying (20–35% of the sample thickness). In the case of samples that were dried in an Environmental Scanning Electron Microscope chamber at 25% humidity, 20 ± 0.2 °C, at 590 Pa, for 5 h, it was noticed that the cracking pattern was characterized by multiple crack cuts at an angle of about 120°. Many dead-end cracks were also found. In case of drying in a climatic chamber at normal atmospheric pressure it was found that the cracks intersections resemble more a 90°–180° system.

Lura et al. [113] studied the geometric features of the cracking pattern in cement paste. The observations were carried out on samples with a diameter of 10 mm, in which there was a centrally located steel rod with a diameter of 1.5 mm, 3 mm or 6 mm. The factor causing the cracking was the autogenous shrinkage of the cement paste. The crack detection was performed by impregnation with galium and analysis was performed by means of an optical and scanning electron microscopy. It was observed that as the thickness of the cement paste envelope around the steel rod increased (a rod diameter decreased), the samples showed the negative fracture geometry, which resulted in a stable crack growth. The length of the crack was greater the smaller the diameter of the rod was used.

Xuan et al. [114] examined the shrinkage cracking characteristics of concrete produced from the construction waste, mainly crushed concrete and masonry aggregates. The influence of four factors, i.e., masonry content, moisture content, degree of compaction and cement content, on the characteristics of the cracking pattern was examined. Two cracks characteristics, i.e., the crack width and the crack spacing, were studied. It was found that the crack width decreases with decreasing amount of cement, decreasing the compaction degree, increasing moisture and increasing the masonry content. In the case of the crack spacing it was found that this parameter decreases with decreasing the amount of cement, increasing the compaction degree, increasing moisture and decreasing the masonry content.

### 5.3. Water Permeability

Wagner et al. [96] made water permeability of strain-hardening cement-based composites dependent on quantitative parameters of the cracking patterns. The samples that were pre-cracked as a result of the uniaxial tension test were tested. To better describe the relationship between the cracking pattern and water permeability a new parameter was introduced, which was called the hydraulic crack pattern parameter *R_WP_*. It was the product of chosen class width, the crack density and weighted crack width value. There was a strong correlation between *R_WP_* and the water flow rate. In the course of the analyses, an equation binding the crack width with water permeability was also formulated for which the Hagen–Poiseuille equation was the starting point.

Lepech and Li [108] studied the effect of cracking degree of high performance fiber reinforced cementitious composites on water permeability. They limited the characteristics of the cracking pattern to determining the maximum crack width and to counting the number of cracks in the analyzed section. They found that with the increase of crack width, the value of the coefficient of permeability also increases. Moreover, normalized permeability per single crack was calculated. It was observed that this parameter also increases with the increase in crack opening width, although the number of cracks in the analyzed section is higher. The study also indicated a beneficial effect of PVA fibers in the context of reducing crack opening width and thus increasing water-tightness of the cement composite.

In the studies carried out by Aldea et al. [109], the relationships between cracks and water permeability of a normal strength concrete were observed. A controlled splitting tensile test was used to introduce cracks into the concrete structure and then the material was tested for water permeability. The results obtained indicated that water permeability increased significantly as the opening width of the cracks increases. Moreover, high repeatability of results for the same cracking levels was obtained. It was also found that the relationship between crack length and water flow is not linear, which was found for the relationship between the crack area and water flow.

An extensive research program was carried out by Torrijos et al. [112]. Two series of ordinary concretes were tested. Within the framework of the conducted works, the influence of the characteristics of the cracking patterns, expressed in the form of crack width and the crack density, was determined on a number of parameters related to permeability, i.e., the water absorption, water penetration, capillary absorption, water permeability. The cracking pattern was created by subjecting the samples to various degradation processes, including: low humidity drying, load at 150 °C and 500 °C and alkali-silica reaction. The highest crack density was found for concretes subjected to 500 °C load, cracks mainly occurred in the interface and to a lesser extent in the cement matrix. In case of samples subjected to drying in low humidity it was found that the velocity and capacity of capillary absorption increased with the crack density. This increase occurred to a certain maximum and then decreased, although the crack width and the crack density continued to increase. For concretes exposed to the alkali-silica reaction a high permeability was initially noticed, followed by a sharp drop after the first few hours. Rehydration of the gel at the crack sites was given as the reason, which resulted in sealing of the structure. The degree of internal destruction of the samples was evidenced by measuring the ultrasonic wave flow rate. A direct relationship between the pulse velocity and the crack density was found.

### 5.4. Mechanical Strength

Fahridzadeh et al. [105] proposed a new approach to assess the degree of degradation of reinforced concrete structures. They proposed to use the fractal dimension to describe the morphology of the cracking pattern on the surface of reinforced concrete. The effect was to develop the damage index, which was calculated according to the equation:

(4) $$DI=\frac{D_{i}-D_{l}}{2-D_{l}}$$

where:

DI—the damage index; 0≤DI≤1,
$D_{i}$—the fractal dimension of the current status of the cracking pattern,
$D_{l}$—the fractal dimension of the cracking pattern computed during the first inspection.

The first inspection shall be considered to be the moment when the appearance of cracks on the surface of the component under test is observed as a result of the applied load. The tests carried out on the reversed cycled loaded reinforced concrete walls indicated high practical suitability of *DI*. It was found that the value of both the fractal dimension and *DI* increases with the next load cycle. It was also shown that *DI* can be used to estimate the remnant lateral stiffness of the wall.

The continuation of the above studies is presented in [115,117]. It was noted that in the literature there are practically no works that would correlate the morphology of the cracking patterns with the structural integrity of the studied material, in this case concrete. The multifractal analysis was used in the study and the considerations were carried out on synthetic crack patterns as well as on two real reinforced concrete walls subjected to cyclic mechanical loading. It was found that the development of cracks structure through an increase in their opening width, length, density, is accompanied by a correlated increase of the multifractal parameters. A rapid increase of these parameters was also observed when the concrete walls tested showed severe stiffness loss.

The subject of Ebrahimkhanlou et al. [118] was the analysis of the cracking patterns on the surface of pre-stressed concrete beams. The analysis was focused on the fractal and multifractal characteristics of cracks patterns, which were formed by a mechanical force. Once again, the high usefulness of the fractal dimension for quantitative description of the degree of development of the cracking patterns was confirmed. Moreover, it was proved that fractal analysis can be used to identify different cracking mechanisms.

Fooladi et al. [119] conducted research to determine the effect of the aggregate grading on the multifractal characteristics of the cracking patterns of concrete, with different compressive strengths. The study was carried out on two series of concretes for which the aggregate grading curve assumed the well-graded and gap-graded characteristics respectively. In the well-graded aggregate samples, it was observed that the value of the fractal dimension reached a saturation level, for which the increase in load did not cause any further change in the value of the fractal dimension. For samples with the gap-graded aggregate a continuous increase in the value of the fractal dimension was observed along with an increase in the value of mechanical load. In the study, the singularity spectrum analysis was also carried out, which, combined with the analysis of the fractal dimension, allowed to conclude that the development of cracks in both series of samples is characterized by multifractal character, but with different intensity. The study concluded that the multifractal approach allows to explain local irregularities of the cracking pattern.

### 5.5. Chemical Corrosion

Xu et al. [9] apart from studying the development of the cracking patterns as a function of the influence of elevated temperature of concrete with fly-ash, also conducted the rapid chloride diffusion test. The results obtained indicated that deterioration of concrete durability is correlated with the crack density.

Zhuang et al. [116] performed a fractal analysis of the crack patterns on the surface of reinforced concrete piles bonded with carbon fiber reinforced polymer. The factor causing the cracks was the corrosion of the reinforcement caused by a harsh marine environment. Within the framework of the research the damage index was developed to assess the degree of degradation of the piles, the method of calculation of which was very similar to the one presented in [105]. The assessment was made at different stages of reinforcement corrosion development. Together with the progressing corrosion, an increase in the value of fractal dimension of the cracking patterns was observed. Moreover, a study of maximum load of piles was carried out, which showed a very high correlation with the fractal dimension. The observed strength loss-fractal dimension dependence assumed a linear dependence. As a result of the conducted tests it was found that supplementing the measurements with the analysis of cracks width, a high quality information could be obtained regarding the degree of structure degradation in the situation when it is impossible to perform mechanical tests.

## 6. Summary

The paper provides a literature review of the development process, analysis and impact of the cracking patterns on the properties of cement composites. The review focuses on four main aspects related to the analysis of the cracking patterns:

- the process of cracks formation in brittle cement composites and their evolution into an extensive and complex system of cracks,
- methods and techniques of digital extraction of the cracking patterns for their further evaluation,
- quantitative parameters used to describe the complexity of the cracking patterns,
- evaluation of the influence of morphology of the cracking patterns on selected properties of cement composites.

Each of these four aspects requires a different scientific approach, making comprehensive analysis of the cracking patterns a difficult and multidisciplinary issue. The three main scientific areas that underpin this research area are the fracture mechanics, image analysis and cement composites technology.

The factors that determine the cracking process, i.e., the number and type of structure defects and the composition of the material, are discussed. It was found that critical stress intensity factor (*K_IC_*) being the product of crack work and Young’s modulus is the most commonly studied parameter which determines the ability of the material to propagate and develop cracks in the branched cracks system. The methodology of analysis of the cracking patterns requires the use of advanced tools for the detection and extraction of cracks. Research carried out so far in this field indicates that by far the most effective and popular group of tools is the computer image analysis. The simplest of them are threshold operations, e.g., the global thresholding, locally adaptive thresholding or the Otsu thresholding. However, such operations, despite the fact that they are simple to implement, are sometimes characterized by a very large detection error. More advanced techniques, such as the genetic algorithms, artificial neural networks or machine learning algorithms, are characterized by very good detection accuracy. Their disadvantage is the need for much more computational resources, which slows down the whole process and makes it sometimes impossible to analyze a large set of data. The literature review shows that the most accurate methods of cracks detection on cement composite surfaces are characterized by an accuracy of more than 95%.

Previous research indicates that the simplest parameter to measure, in terms of the complexity and morphology of the cracking patterns, is the width of the crack opening. However, this indicator does not provide information on the layout and organization of the cracks structure in the material. The parameter that provides this information is the crack density and the resultant the crack spacing. Recent studies postulate the use of fractal geometry to quantify the cracking patterns. In these studies, the concept of fractal dimension is used, which by definition determines the degree of complexity of the analyzed structure. Analyses carried out with the use of this parameter indicate a strong relationship with the mechanical features of a degraded cement composite. In the research results published so far, the greatest attention is paid to determining the influence of the cracking patterns on the performance of cement composites at elevated temperatures. The system of cracks formed due to the shrinkage and drying of the material as well as those formed due to chemical corrosion is also analyzed. The influence of the cracking patterns on water permeability and mechanical strength of cement composites is also evaluated. The presented results indicate that with the progressive development of the cracking patterns, practically every property of the hardened cement composite deteriorates.

The authors of many research point to the key importance of the degree of development of the cracking patterns for the functional properties and durability of cement composites. However, there are still few works in which the dependence of the cracking patterns on the physical and mechanical properties of the material is determined directly, in a numerical way. Knowledge in this area seems to be crucial in the aspect of designing durable cement composites, which would be resistant to cracking and crack development process in specific environments. From the methodological point of view, further development of works aimed at developing more and more accurate, automatic crack detection systems is indicated. Such systems could be used very successfully by building managers to effectively detect areas in need of remedial action. The search for new methods and the development of the relationship between the cracking patterns and material properties is crucial in the development of non-destructive testing methods in cement composite technology.

## Figures and Tables

**Table 1:** Types of cracks characteristic for structural elements made of cement-based composites (according to [52]).

Type of Cracks	Reasons	Occurence Time	Typical Location
plastic settlement	excessive secretion of cement milk—bleeding; rapid initial drying	10 min–3 h	sections of considerable depth; top of column, trough and coffered panels
plastic shrinkage	rapid initial drying; reinforcement at the surface; low speed of drained water discharge	30 min–6 h	surfaces; slabs
early thermal contraction	excessive heat generation; excessive temperature gradients; rapid cooling	1 day–(2–3 weeks)	thick walls; thick plates
prolonged shrinkage during drying	ineffective connections; excessive contraction; inadequate care	several weeks or months	thin panels; walls
map-cracking	excessive rubbing; rich mix; inadequate care	1–7 days	walls; plates
corrosion of the reinforcement	insufficient cover; poor concrete quality	over 2 years	columns; beams
alkaline reactions in the aggregate	reactive aggregate; cement with high alkali content	over 5 years	moistened elements
ankle cracks	aggregates damaged by frost	over 10 years	free ends of plates

**Table 2:** A summary of the cracks identification techniques and their accuracy, with literature references.

Digital Image Processing Technique	References	Accuracy Level (Only Where the Authors Have Given It)
Global thresholding	[54,55,56,84]	≤90% [54]
Locally adaptive thresholding	[55,56,57,58,59,60]	90–95% [58]
Otsu thresholding	[61,62,63,64,73]	≥95% [63]
Edge detection	[54,63,71]	≤90% [54]; 90–95% [71]; ≥95% [63]
Probabilistic approach	[55]	-
Median filtering	[55]	-
Sobel’s filtering	[62]	-
Morphological approach	[57,58,59,77,81]	90–95% [58]; 90–95% [77]
Genetic algorithms	[65,67,68,69]	≤90% [67]
Fuzzy logic based techniques	[70,71,72,73]	90–95% [71]
Artificial neural networks	[71,75,76,77,78,79,80,81]	≤90% [76,78]; 90–95% [71,77]; ≥95% [75,79,80]
Wavelet approach	[76]	≤90% [76]
Dijkstra algorithm	[83,84,85,86]	-
The Bayesian classifier	[88,89,90,91,92]	90–95% [90]
The AdaBoost classifier	[94,95]	≤90% [95]
Statistical methods	[89]	-

**Table 3:** Analysis by parameter used, with references to the literature.

Parameter	References
Opening width of the cracks	[9,12,13,14,15,16,96,97,100,101,102,105,106,107,108,109,110,111,112,113,114,115,116]
Length and orientation of the cracks	[8,12,13,14,15,16,98,99,107,109,112,113]
The crack density	[8,9,13,96,99,100,110,111,112,114]
The crack area	[12,13,14,15,16,98,101,102,106,107,109]
The fractal dimension	[105,115,116,117,118,119]

## References

[1] Neville A.M. (2000). Properties of Concrete.

[2] Brandtzaeg A. (1927). Failure of A Material Composed of Non-isotropic Elements.

[3] Yoshida H. (1930). Über Das Elastische Verhalten Von Beton: mit Besonderer Berücksichtigung Der Querdehnung.

[4] VanMier J. (2013). Concrete Fracture: A Multiscale Approach.

[5] Recho N. (2012). Fracture Mechanics and Crack Growth.

[6] Kurumatani M., Terada K., Kato J., Kyoya T., Kashiyama K. (2016). An isotropic damage model based on fracture mechanics for concrete. Eng. Fract. Mech..

[7] Hlobil M., Smilauer V., Chanvillard G. (2016). Micromechanical multiscale fracture model for compressive strength of blended cement pastes. Cem. Concr. Res..

[8] Bisschop J., Wittel F. (2011). Contraction gradient induced microcracking in hardened cement paste. Cem. Concr. Compos..

[9] Xu Y., Wong Y., Poon C., Anson M. (2003). Influence of PFA on cracking of concrete and cement paste after exposure to high temperatures. Cem. Concr. Res..

[10] Tosun-Felekoglu K., Felekoglu B. (2013). Effects of fibre hybridization on multiple cracking potential of cement-based composites under flexural loading. Constr. Build. Mater..

[11] Banthia N., Azzabi M., Pigeon M. (1993). Restrained shrinkage cracking in fiber-reinforced cementitious composites. Mater. Struct..

[12] Szelag M. (2019). Evaluation of cracking patterns of cement paste containing polypropylene fibers. Compos. Struct..

[13] Szelag M. (2019). Properties of cracking patterns of multi-walled carbon nanotube-reinforced cement matrix. Materials.

[14] Szelag M. (2018). Development of Cracking Patterns in Modified Cement Matrix with Microsilica. Materials.

[15] Szelag M. (2018). Influence of specimen’s shape and size on the thermal cracks’ geometry of cement paste. Constr. Build. Mater..

[16] Szelag M. (2018). The Influence of Metakaolinite on the Development of Thermal Cracks in a Cement Matrix. Materials.

[17] Griffith A. (1921). The phenomena of rupture and flow in solids. Philos. Trans. R. Soc. Lond. Sereis A.

[18] Mindess S., Barnes P. (1983). Structure and Performance of Cements.

[19] Mindess S. (1984). Fracture toughness testing of cement and concrete. Fracture Mechanics of Concrete: Material Characterization and Testing.

[20] Powers T.C. (1968). Rheology of Freshly Mixed Concrete. The Properties of Fresh Concrete.

[21] Oberholster R.E. Pore structure, permeability and diffusivity of hardened cement paste and concrete in relation to durability: Status and prospects. Proceedings of the 8th International congress on chemistry of cement.

[22] Brown J.H., Pomeroy C.D. (1973). Fracture toughness of cement paste and mortars. Cem. Concr. Res..

[23] Hillemeier B., Hilsdorf H. (1977). Fracture mechanics studies on concrete compounds. Cem. Concr. Res..

[24] Beaudoin J. (1982). Effect of humiidty and porosity on fracture of hardened Portland-cement. Cem. Concr. Res..

[25] Shah S. (1990). Determination of fracture parameters (KIcs and CTODc) of plain concrete using three-point bend tests. Mater. Struct..

[26] Chen X., Wu S., Zhou J. (2013). Influence of porosity on compressive and tensile strength of cement mortar. Constr. Build. Mater..

[27] Kumar R., Bhattacharjee B. (2003). Porosity, pore size distribution and in situ strength of concrete. Cem. Concr. Res..

[28] Lian C., Zhuge Y., Beecham S. (2011). The relationship between porosity and strength for porous concrete. Constr. Build. Mater..

[29] Roy D., Gouda G. (1973). Porosity-strength relation in cementitious materials with very high strengths. J. Am. Ceram. Soc..

[30] Taylor H. (1977). Discussion of the paper “Microstructure and strength of hydrated cements” by RF Feldman and JJ Beaudoin. Cem. Concr. Res..

[31] Mindess S. (1970). Relation between the compressive strength and porosity of autoclaved calcium silicate hydrates. J. Am. Ceram. Soc..

[32] Nadeau J., Mindess S., Hay J. (1974). Slow crack growth in cement paste. J. Am. Ceram. Soc..

[33] Wittmann F. (1979). Trends in research on creep and shrinkage of concrete. Cement Production an Use.

[34] Birchall J., Howard A., Kendall K. (1981). Flexural strength and porosity of cements. Nature.

[35] Alford N., Rahman A. (1981). An assesment of porosity and pore sizes in hardened cement pastes. J. Mater. Sci..

[36] Wittmann F. (1968). Surface tension skrinkage and strength of hardened cement paste. Matériaux et Construction.

[37] Robertson B., Mills R. (1985). Influence of sorbed fluids on compressive strength of cement paste. Cem. Concr. Res..

[38] Costa U., Massazza F. (1986). Rheological properties of fly ash cement pastes. Il Cemento.

[39] Beaudoin J. (2000). Calcium hydroxide in cement matrices: Physico-mechanical and physico-chemical contributions. Calcium Hydroxide in Concrete in Materials Science of Concrete.

[40] Alford N., Groves G., Double D. (1982). Physical properties of high-strength cement pastes. Cem. Concr. Res..

[41] Neville A. (1959). The influence of the direction of loading on the strength of concrete test cubes. ASTM Bull..

[42] Hsu T., Slate F., Sturman G., Winter G. (1963). Microcracking of plain concrete and the shape of the stress-strain curve. J. Am. Concr. Inst..

[43] Akcaoglu R., Tokyay M., Celik T. (2005). Assessing the ITZ microcracking via scanning electron microscope and its effect on the failure behavior of concrete. Cem. Concr. Res..

[44] Golewski G. (2018). An assessment of microcracks in the Interfacial Transition Zone of durable concrete composites with fly ash additives. Compos. Struct..

[45] Jornet A., Guidali E., Muhlethaler U. (1993). Microcracking in high-performance concrete. Proceedings of the 4th euroseminar on microscopy applied to building materials.

[46] Slate F., Hover K. (1984). Microcracking in concrete. Fracture Mechanics of Concrete: Material Characterization and Testing.

[47] Smadi M., Slate F. (1989). Microcracking of high and normal strength concreters under short-term and long-term loadings. Mater. J..

[48] Solomatov V.I. (2001). Polystructural theory development of composite building materials. The Success of Modern Materials Science. Proceedings of the Anniversary Conference, RAASN.

[49] Solomatov V.I., Vyrovoy V.N., Bobryshev A.N. (1991). Polystructural Theory of Composite Building Materials.

[50] Sukhanov V., Vyrovoy V., Dorofeev V. (2011). Crack’s role in structural development of the constructional composite materials. Mod. Ind. Civ. Constr..

[51] Vyrovoy V.N., Dorofeev V.S., Sukhanov V.G. (2010). Composite Building Materials and Constructions. Structure, Self-Organization, Properties.

[52] Report C.S. (1992). Non-Structural Cracks in Concrete. Technical Report no. 22.

[53] Henrichsen A., Laugesen P., Diamond S., Mindess S., Glasser F., Roberts L., Skalny J., Wakeley L. (1995). Monitoring of concrete quality in high performance civil engineering constructions. MRS Online Proceed. Library Arch..

[54] Acosta J.A., Figueroa J.L., Mullen R.L. (1992). Low-cost video image processing system for evaluating pavement surface distress. Transp. Res. Rec..

[55] Fujita Y., Hamamoto Y. (2011). A robust automatic crack detection method from noisy concrete surfaces. Mach. Vis. Appl..

[56] Sezgin M., Sankur B. (2004). Survey over image thresholding techniques and quantitative performance evaluation. J. Electron. Imag..

[57] Tang J., Gu Y. Automatic crack detection and segmentation using a hybrid algorithm for road distress analysis. Proceedings of the 2013 IEEE International Conference on Systems, Man, and Cybernetics.

[58] Tong X., Guo J., Ling Y., Yin Z. (2001). A new image-based method for concrete bridge bottom crack detection. Proceedings of the International Conference on Image Analysis and Signal Processing.

[59] Liu X., Ai Y., Scherer S. (2017). Robust image-based crack detection in concrete structure using multi-scale enhancement and visual features. Proceedings of the 2017 IEEE International Conference on Image Processing (ICIP).

[60] Gavilan M., Balcones D., Marcos O., Llorca D., Sotelo M., Parra I., Ocana M., Aliseda P., Yarza P., Amirola A. (2011). Adaptive Road Crack Detection System by Pavement Classification. Sensors.

[61] Otsu N. (1979). A threshold selection method from gray-level histograms. IEEE Trans. Syst. Man Cybern..

[62] Talab A., Huang Z., Xi F., Liu H. (2016). Detection crack in image using Otsu method and multiple filtering in image processing techniques. Optik.

[63] Valenca J., Dias-da-Costa D., Julio E. (2012). Characterisation of concrete cracking during laboratorial tests using image processing. Constr. Build. Mater..

[64] Hoang D. (2018). Detection of Surface Crack in Building Structures Using Image Processing Technique with an Improved Otsu Method for Image Thresholding. Adv. Civ. Eng..

[65] Nishikawa T., Yoshida J., Sugiyama T., Saito S., Fujino Y. (2007). Robust Image Procesing for detection of Concrete Cracks Using a Parallel Image-Filter. J. JSCE.

[66] Aoki S., Nagao T. (1999). Automatic construction of tree-structural image transformations using genetic programming. Proceedings of the 10th International Conference on Image Analysis and Processing.

[67] Nishikawa T., Yoshida J., Sugiyama T., Fujino Y. (2012). Concrete Crack Detection by Multiple Sequential Image Filtering. Comput. Aided Civ. Infrastruct. Eng..

[68] Tomikawa T. (1999). A study of road crack detection by the meta-genetic algorithm. Proceedings of the 1999 IEEE Africon. 5th Africon Conference in Africa (Cat. No. 99CH36342).

[69] Medina R., Llamas J., Gomez-Garcia-Bermejo J., Zalama E., Segarra M. (2017). Crack Detection in Concrete Tunnels Using a Gabor Filter Invariant to Rotation. Sensors.

[70] Zadeh L. (1988). Fuzzy-logic. Computer.

[71] Choudhary G., Dey S. (2012). Crack Detection in Concrete Surfaces using Image Processing, Fuzzy Logic, and Neural Networks. Proceedings of the 2012 IEEE Fifth International Conference on Advanced Computational Intelligence (ICACI).

[72] Cheng H., Chen J., Glazier C., Hu Y. (1999). Novel approach to pavement cracking detection based on fuzzy set theory. J. Comput. Civ. Eng..

[73] Maode Y., Shaobo B., Xue L., Yuyao H. (2007). An adaptive fuzzy image enhancement algorithm for local regions. Proceedings of the 2007 Chinese Control Conference.

[74] Haykin S. (1994). Neural Networks: A Comprehensive Foundation.

[75] Lee B., Lee H. (2004). Position-invariant neural network for digital pavement crack analysis. Comput. Aided Civ. Infrastruct. Eng..

[76] Bray J., Verma B., Li X., He W. (2006). A neural network based technique for automatic classification of road cracks. Proceedings of the 2006 IEEE International Joint Conference on Neural Network Proceedings.

[77] Moon H., Kim J. Intelligent crack detecting algorithm on the concrete crack image using neural network. Proceedings of the 28th ISARC.

[78] Dung C., Anh L. (2019). Autonomous concrete crack detection using deep fully convolutional neural network. Autom. Constr..

[79] Zhang J., Lu C., Wang J., Wang L., Yue X. (2019). Concrete Cracks Detection Based on FCN with Dilated Convolution. Appl. Sci..

[80] Cha Y., Choi W., Caicedo J., Pakzad S. (2017). Vision-Based Concrete Crack Detection Using a Convolutional Neural Network. Dyn. Civ. Struct..

[81] Lee B., Kim Y., Yi S., Kim J. (2013). Automated image processing technique for detecting and analysing concrete surface cracks. Struct. Infrastruct. Eng..

[82] Dijkstra E. (1959). A note on two problems in connexion with graphs. Numer. Math..

[83] Amhaz R., Chambon S., Idier J., Baltazart V. (2016). Automatic Crack Detection on Two-Dimensional Pavement Images: An Algorithm Based on Minimal Path Selection. IEEE Trans. Intell. Transp. Syst..

[84] Gunkel C., Stepper A., Muller A., Muller C. (2012). Micro crack detection with Dijkstra’s shortest path algorithm. Mach. Vis. Appl..

[85] Yu S., Jang J., Han C. (2007). Auto inspection system using a mobile robot for detecting concrete cracks in a tunnel. Autom. Constr..

[86] Lee S.Y., Lee S., Shin D., Son Y., Han C. (2007). Development of an inspection system for cracks in a concrete tunnel lining. Can. J. Civ. Eng..

[87] Lindley D. (2005). Kendall’s advanced theory of statistics, volume 2B, Bayesian inference, 2nd edition. J. R. Stat. Soc. Ser. A Stat. Soc..

[88] Schmugge S., Nguyen N., Thao C., Lindberg J., Grizzi R., Joffe C., Shin M. Automatic detection of cracks during power plant inspection. Proceedings of the 3rd International Conference on Applied Robotics for the Power Industry.

[89] Hutchinson T., Chen Z. (2006). Improved image analysis for evaluating concrete damage. J. Comput. Civ. Eng..

[90] Valenca J., Goncalves L., Julio E. (2013). Damage assessment on concrete surfaces using multi-spectral image analysis. Constr. Build. Mater..

[91] Oliveira H., Correia P.L. (2015). Supervised strategies for cracks detection in images of road pavement flexible surfaces. Proceedings of the 2008 16th European Signal Processing Conference.

[92] Oliveira H., Correia P. Automatic Crack Pavement Detection Using a Bayesian Stochastic Pattern Recognition System. Proceedings of the RECPAD2007.

[93] Freund Y., Schapire R., Abe N. (1999). A short introduction to boosting. J. Jpn. Soc. Artif. Intell..

[94] Cord A., Chambon S. (2012). Automatic Road Defect Detection by Textural Pattern Recognition Based on AdaBoost. Comput. Aided Civ. Infrastruct. Eng..

[95] Prasanna P., Dana K., Gucunski N., Basily B., La H., Lim R., Parvardeh H. (2016). Automated Crack Detection on Concrete Bridges. IEEE Trans. Autom. Sci. Eng..

[96] Wagner C., Villmann B., Slowik V., Mechtcherine V. (2017). Water permeability of cracked strain-hardening cement-based composites. Cem. Concr. Compos..

[97] Wagner C., Dollase A., Slowik V. Evaluation of crack patterns in SHCC with respect to water permeability and capillary suction. Proceedings of the 3rd International Conference on Concrete Repair, Rehabilitation and Retrofitting (ICCRRR).

[98] Fic S., Szelag M. (2015). Analysis of the development of cluster cracks caused by elevated temperatures in cement paste. Constr. Build. Mater..

[99] Mobasher B., Stang H., Shah S. (1990). Microcracking in fiber reinforced-concrete. Cem. Concr. Res..

[100] Magalhaes M., Toledo R., Fairbairn E. (2015). Thermal stability of PVA fiber strain hardening cement-based composites. Constr. Build. Mater..

[101] Szelag M., Szewczak A. (2019). Evaluation of Dependencies between Physico-Mechanical Properties and the Thermal Cracks’ Geometry of Cement Pastes Modified with Metakaolinite Using the LSM Method. 3rd World Multidisciplinary Civil Engineering, Architecture, Urban Planning Symposium (WMCAUS 2018). IOP Conf. Ser. Mater. Sci. Eng..

[102] Szeląg M., Szewczak A. (2019). Dependencies between Cracking Patterns and the Physico-Mechanical Properties of Microsilica Modified Cement Matrix. In Proceedings of IOP Conference Series: Mater. Sci. Eng..

[103] Mandelbrot B.B. (1983). The Fractal Geometry of Nature.

[104] Mandelbrot B.B. (1977). Fractals: Form, Chance, and Dimension.

[105] Farhidzadeh A., Dehghan-Niri E., Moustafa A., Salamone S., Whittaker A. (2013). Damage Assessment of Reinforced Concrete Structures Using Fractal Analysis of Residual Crack Patterns. Exp. Mech..

[106] Wang K., Shah S., Phuaksuk P. (2001). Plastic shrinkage cracking in concrete materials–Influence of fly ash and fibers. Mater. J..

[107] Bakhshi M., Mobasher B. (2011). Experimental observations of early-age drying of Portland cement paste under low-pressure conditions. Cem. Concr. Compos..

[108] Lepech M., Li V. (2009). Water permeability of engineered cementitious composites. Cem. Concr. Compos..

[109] Aldea C., Ghandehari M., Shah S., Karr A. Combined effect of cracking and water permeability of concrete. Proceedings of the 14th Engineering Mechanics Conference.

[110] Bazant Z., Raftshol W. (1982). Effect of cracking in drying and shrinkage specimens. Cem. Concr. Res..

[111] Bazant Z., Sener S., Kim J. (1987). Effect of cracking on drying permeability and diffusivity of concrete. Mater. J..

[112] Torrijos M., Giaccio G., Zerbino R. (2010). Internal cracking and transport properties in damaged concretes. Mater. Struct..

[113] Lura P., Jensen O., Weiss J. (2009). Cracking in cement paste induced by autogenous shrinkage. Mater. Struct..

[114] Xuan D., Molenaar A., Houben L. (2016). Shrinkage cracking of cement treated demolition waste as a road base. Mater. Struct..

[115] Ebrahimkhanlou A., Farhidzadeh A., Salamone S. (2016). Multifractal analysis of crack patterns in reinforced concrete shear walls. Struct. Health Monit. Int. J..

[116] Zhuang N., Dong H., Zhou Y., Chen D. (2018). Cracking behavior of reinforced concrete piles externally bonded with carbon fiber reinforced polymer in a marine environment. Constr. Build. Mater..

[117] Ebrahimkhanlou A., Farhidzadeh A., Salamone S., Lynch J., Wang K., Sohn H. (2015). Multifractal analysis of two-dimensional images for damage assessment of reinforced concrete structures. Sens. Smart Struct. Techn. Civil Mech. Aeros. Syst..

[118] Ebrahimkhanlou A., Athanasiou A., Hrynyk T., Bayrak O., Salamone S. (2019). Fractal and Multifractal Analysis of Crack Patterns in Prestressed Concrete Girders. J. Bridge Eng..

[119] Fooladi A., Banan M. (2016). Multifractal Analysis of Crack Propagation in Concrete Specimens Considering the Influence of the Aggregates’ Grading. Iran. J. Sci. Technol.-Trans. Civ. Eng..

[120] Kim K., Yun T., Park K. (2013). Evaluation of pore structures and cracking in cement paste exposed to elevated temperatures by X-ray computed tomography. Cem. Concr. Res..

